# Facile One-Step Hydrothermal Fabrication of (Sr_0.6_Bi_0.305_)_2_Bi_2_O_7_/SnO_2_ Heterojunction with Excellent Photocatalytic Activity

**DOI:** 10.3390/nano10020321

**Published:** 2020-02-13

**Authors:** Di Zhu, Xinling Wang, Huiting An, Yan Zhong, Dianhui Wang, Chengying Tang, Chaohao Hu

**Affiliations:** 1 School of Materials Science and Engineering, Guilin University of Electronic Technology, Guilin 541004, China; heyjude456@163.com (D.Z.); xlwang04@163.com (X.W.); ahq1399615134@163.com (H.A.); zyguet@163.com (Y.Z.); dhwang@guet.edu.cn (D.W.); ctang@guet.edu.cn (C.T.); 2 Guangxi Key Laboratory of Information Materials, Guilin University of Electronic Technology, Guilin 541004, China

**Keywords:** (Sr_0.6_Bi_0.305_)_2_Bi_2_O_7_, (Sr_0.6_Bi_0.305_)_2_Bi_2_O_7_/SnO_2_, hydrothermal method, heterojunction, photocatalytic activity

## Abstract

The pyrochlore-type (Sr_0.6_Bi_0.305_)_2_Bi_2_O_7_ (SBO), containing Bi^3+^ and Bi^5+^ mixed valent states, was recently found to be used as a new visible light responsive photocatalyst. Novel SBO/SnO_2_ heterostructured composites were synthesized through a facile one-step hydrothermal method. The phase structure, morphology, chemical composition, and optical properties of the obtained samples were characterized by XRD, SEM, TEM, XPS, and UV-vis DRS. Compared to pure SBO and SnO_2_, the synthesized SBO/SnO_2_ composites exhibited significantly enhanced photocatalytic efficiency. The results indicated that the photoinduced holes and superoxide radicals play a dominant role and are the main reactive species during the degradation of Methylene Blue (MB) solution under visible light irradiation. Heterojunctions, formed in samples, directly contribute to the improvement of photocatalytic efficiency of SBO/SnO_2_ composites, since it not only broadens the light response range, but also accelerates the separation of photogenerated carriers.

## 1. Introduction

Semiconductor-based photocatalysis has been considered one effective strategy to deal with global environmental pollution and the energy crisis [1,2,3,4]. TiO_2_ has been extensively studied as a popular photocatalyst since the photocatalytic splitting of water on TiO_2_ photochemical electrodes was first reported in 1972 [5]. However, the relatively large band gap of about 3.2 eV for the anatase TiO_2_ and low quantum efficiency directly limit its wider practical applications [6]. In recent years, many researchers have devoted a lot of effort to developing new visible light responsive photocatalysts [7,8,9,10,11].

Recently, a series of bismuth-based semiconductor photocatalysts have drawn much attention, owing to their special crystal structure and electronic structure. Many Bi^3+^-containing semiconductor compounds like Bi_2_O_3_ [12], Bi_2_WO_6_ [13], BiVO_4_ [14], BiOX (X = Cl, Br, I) [15,16], Bi_2_SnO_7_ [17], and Bi_2_O_2_CO_3_ [18] have been found to exhibit high photocatalytic activity, driven by visible light, owing to the hybridization of O-2*p* and filled Bi-6*s* orbitals. Some Bi^5+^-containing compounds such as NaBiO_3_ [19], KBiO_3_ and MgBi_2_O_6_ [20] also possess excellent photocatalytic performance due to the contribution from the Bi-5*d* and O-2*p* orbitals. In addition, recent studies [21,22] have indicated that Bi_2_O_4_ containing the mixed valent states of Bi (Bi^3+^ and Bi^5+^) can serve as an important visible light responsive photocatalyst with much higher photocatalytic degradation activity in comparison to some well-investigated, highly efficient photocatalysts.

In fact, (Sr_0.6_Bi_0.305_)_2_Bi_2_O_7_ (SBO) can also be considered one kind of special Bi-based oxides with Bi^3+^ and Bi^5+^ mixed valent states. Moreover, it possesses the cubic pyrochlore-type structure similar to that of Bi_2_Sn_2_O_7_. In SBO, some Bi^3+^ and Bi^5+^ ions locate at the 16*c* sites (Wyckoff representation), Sr^2+^ and another part of Bi^3+^ ions, as well as a small amount of vacancies that occupy the 16d sites. While the synthesis and structural characterization of SBO have been previously reported [23], its chemical catalytic property has not attracted enough attention to date. Very recently, we have successfully fabricated the SBO compound via the hydrothermal method and found that SBO can photodegrade various organic dyes and possesses visible light photocatalytic activity [24]. However, the band gap of SBO is relatively small at about 1.25 eV, and quick recombination of photoinduced electrons and holes readily occur, undoubtedly affecting its photocatalytic efficiency [25,26].

Currently, the construction of heterojunctions with multiple functional components has been considered to be an extremely effective pathway to improve the photocatalytic activity of semiconductor photocatalysts, since not only the light absorption in the heterostructured composites is significantly enhanced, but the separation and transportation of photogenerated charge carriers are also effectively promoted [27,28,29]. Tin dioxide (SnO_2_) has a relatively large band gap of about 3.8 eV and fails to absorb light with wavelengths over 330 nm [29]. However, a great amount of research on SnO_2_-related heterojunctions, via coupling with other semiconductors like TiO_2_ [30], ZnO [31], BiOI [32], and NiO [33], has verified the significantly enhanced photocatalytic activity of composites.

In this work, we devoted a lot of effort to synthesizing the SBO/SnO_2_ heterostructured composites through a facile one-step hydrothermal method. The phase structure, morphology, microstructure, and optical property of the as-prepared composites were characterized by various experimental techniques. The photocatalytic activity of SBO/SnO_2_ heterojunctions driven by visible light was estimated by the photochemical degradation of MB dye. Moreover, the mechanism for the enhancement of photocatalytic efficiency for the composite catalysts was proposed by trapping the free radicals’ experiments and relative photoelectrochemical measurements.

## 2. Materials and Methods

### 2.1. Materials

The chemicals for synthesis were used in this paper with an analytical purity, and were purchased from Beijing Chemical Reagents Industrial Company of China. All the solutions were prepared with deionized water.

### 2.2. Preparation of Photocatalysts

In this paper, a facile one-step hydrothermal method for the synthesis of the SBO/SnO_2_ composite samples was used. 0.5 mmol of NaBiO_3_·2H_2_O and 1 mmol of Sr(NO_3_)_2_ were, respectively, dissolved in 30 mL of deionized water under vigorous stirring for 10 min at room temperature to prepare solution A and B, respectively. Brown-yellow solution C was further obtained by mixing solution A and B, and by being stirred for about 30 min at room temperature. Subsequently, different contents (1.46 g, 1.21 g, 1.04 g, 0.81 g and 0.728 g) of SnO_2_ were, respectively, added in the five identical portions of solution C, and the pH value was adjusted to 12 by adding 4 M NaOH. The mixture solution was then transferred to a 100 mL of a Teflon-lined stainless autoclave and heated at 140 °C for 6 h in a drying oven. After the hydrothermal reaction, the as-obtained sample was filtered, washed several times with deionized water and absolute ethanol, and finally dried at 60 °C for 12 h. The resultant composites were denoted as 10 wt% SBO/SnO_2_, 12 wt% SBO/SnO_2_, 14 wt% SBO/SnO_2_, 18 wt% SBO/SnO_2_ and 20 wt% SBO/SnO_2_ with the color change from light gray to dark gray, respectively. Pure SBO was also prepared by the same process as the preparation of the samples without SnO_2_.

### 2.3. Characterization

A powder X-ray diffractometer (XRD, Bruker D8-Advance, Germany) was used to characterize the crystal structure of as-synthesized samples. A field-emission scanning electron microscope (FE-SEM, Hitachi, Tokyo, Japan) with an energy dispersive X-ray spectroscopy (EDS) was used to characterize the morphologies and elemental analyses of samples. The microstructures of the samples were further measured on the transmission electron microscopy (TEM, FEI Tecnai G20, Hillsboro, OR, USA), operating at 200 kV. The X-ray photoelectron spectroscopy (XPS) measurement was carried out by using an Escalab 250Xi system with a Al Kα radiation source (1486.6 eV). Fourier transform infrared (FT-IR) spectra were recorded on a FTIR spectrometer (Thermo Fisher Nicolet 6700, Waltham, MA, USA) using the standard KBr disk method. The UV-Vis diffuse reflectance spectra (UV-Vis DRS, Puxi TU-1901, Beijing, China) of the as-prepared photocatalysts were measured with a UV-Vis spectrometer (Puxi TU-1901, Beijing, China), in the range of 230–750 nm, using BaSO_4_ as a reference.

### 2.4. Determination of Photocatalytic Activity

The photocatalytic performance of the obtained samples was evaluated by degrading the MB solution under visible light irradiation. A 300 W xenon lamp was used as the light source, and a UV-cut filter was used to remove the wavelength of light below 400 nm. In detail, 0.2 g of the powdered catalyst was dispersed into 100 mL of MB aqueous solution (10 mg/L) in a quartz beaker. Before irradiation, the resultant mixture was stirred in the dark for 30 min in order to achieve an adsorption–desorption equilibrium between the MB dye and photocatalysts. 3 mL of the mixture was collected at regular intervals and was immediately filtered to remove the catalysts. Subsequently, the remnant concentration of MB at different intervals was determined by measuring the photocatalytic degradation ratio using a Puxi TU-1901 UV-Vis spectrophotometer.

### 2.5. Photoelectrochemical Measurement

The measurement of electrochemical impedance spectra (EIS) for the as-prepared photocatalysts was performed on an electrochemical workstation (Parstat MC, Princeton Applied Research, Delaware, DE, USA) in a conventional three-electrode cell, with an Ag/AgCl electrode, the prepared samples, and a Pt plate as the reference electrode, the working electrode, and the counter electrode, respectively. An aqueous solution containing 0.5 M KOH was chosen as the electrolyte. A 300 W xenon lamp (CEL-HXF300, Aulight, Beijing, China), equipped with an AM 1.5G filter, was used as the visible light source. Nyquist plots were measured in the frequency range from 0.1 Hz to 100 kHz with a voltage amplitude of 10 mV. The transient photocurrent response of samples was measured on a Keysight B1500A semiconductor device analyzer and an Advanced PW-600 probe station. The waveform generator was used to monitor the pulse frequency and optical power determined by an Ophir Nova II handheld laser power/energy meter.

## 3. Results and Discussion

### 3.1. Phase Structure and Chemical Compositions

**Figure 1:**
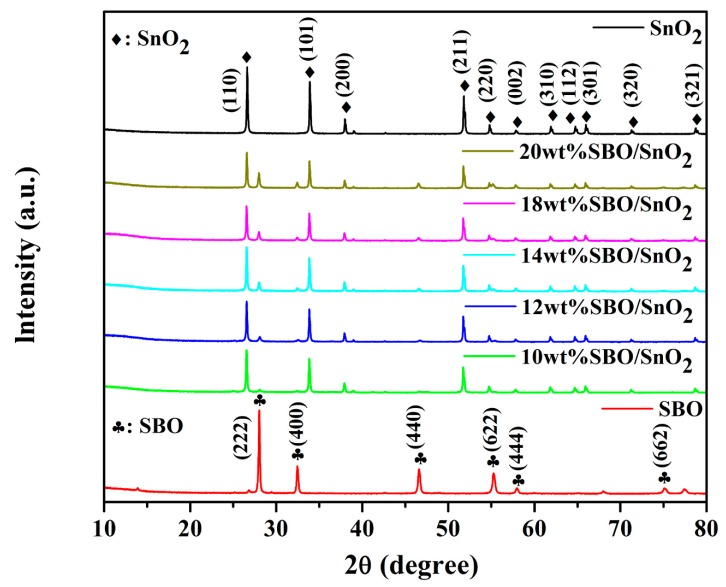
XRD patterns of SnO_2_, SBO, and SBO/SnO_2_ composites containing 5 wt%, 10 wt%, 14 wt%, 18 wt%, and 20 wt% of SBO, respectively.

Figure 1 presents the XRD patterns of as-synthesized SBO, SnO_2_, and SBO/SnO_2_ nanocomposites with different mass ratios. The typical diffraction peaks at 2θ = 26.5°, 33.8°, 37.9°, 51.7°, 54.7°, 57.8°, 61.9°, 64.7°, 65.9° and 78.6° can be readily assigned to the standard card of tetragonal SnO_2_ (JCPDS no. 99-0024). The characteristic diffraction peaks at 2θ = 28.1°, 32.5°, 46.6°, 55.3°, 58.0°, and 75.2° are well indexed to the (222), (400), (440), (622), (444), and (662) lattice planes of the pyrochlore-type SBO (JCPDS card no. 81-2460). The diffraction peaks of SBO and SnO_2_ can be observed in the XRD patterns of SBO/SnO_2_ composites. The sharp and narrow peaks indicate that the obtained SBO/SnO_2_ composites have high crystallinity. Moreover, no other peaks from possible impurities are detected in these patterns. It can also be found that, when the content of SBO is about 10 wt%, the diffraction peaks of SBO are not obvious in the 10 wt% SBO/SnO_2_ composite, except the peak corresponding to the (400) lattice plane, which can be observed. As the concentration of SBO further increases in the composites, the characteristic diffraction peaks of SBO become stronger. However, the measured XRD patterns show that the intensity of all the peaks of SnO_2_ in the composites is decreased gradually by further increasing the content of SBO to higher than 14 wt%.

**Figure 2:**
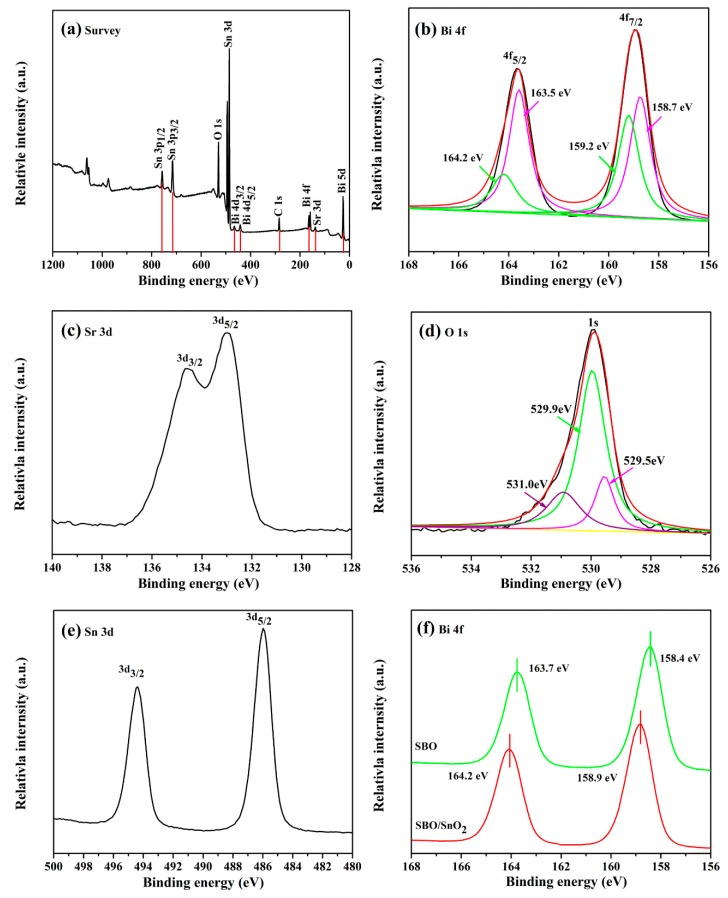
XPS spectra of 14 wt% SBO/SnO_2_ composite: (**a**) full scan survey of all elements and high-revolution spectra of (**b**) Bi 4f, (**c**) Sr 3d, (**d**) O 1s, (**e**) Sn 3d, and (**f**) Bi 4f of pure SBO for comparison.

The measured XPS shown in Figure 2 is used to further investigate the oxidation state and surface chemical compositions of as-prepared composites. The full scan spectrum presented in Figure 2a reveals the presence of Bi, O, Sr, and Sn elements in the as-prepared samples. The peak of C1s at 284.6 eV in the spectrum was assigned to the adhesive hydrocarbons from the XPS instrument itself. The Bi 4f spectra of 14 wt% SBO/SnO_2_ shown in Figure 2b indicate that the peak of Bi 4f_7/2_ (or Bi 4f_5/2_) can be well deconvoluted into two peaks at binding energy of 158.7 and 159.2 eV [34] (or at 163.5 and 164.2 eV), which is attributed to Bi^3+^ and Bi^5+^ [35] and accords well with the previous results on the Bi_2_O_4_-based systems [36,37]. The peaks at binding energy of 132.9 and 134.7 eV shown in Figure 2c are, respectively, assigned to Sr 3d_5/2_ and Sr 3d_3/2_ of Sr^2+^ [38]. For the spectrum of O 1s presented in Figure 2d, the binding energy around 529.5 eV corresponds to the lattice oxygen of Sr-O or Bi-O bonds, which is comparable to our previous measurements on Bi_2_Sn_2_O_7_ and MgBi_2_O_6_ [39]. The peaks at 531.0 and 529.9 eV are ascribed to the physically adsorbed oxygen and chemisorbed oxygen, respectively [40]. The characteristic XPS peaks of Sn 3d presented in Figure 2e are located at 494.4 and 486.0 eV and correspond to Sn 3d_3/2_ and Sn 3d_5/2_ of Sn^4+^. In addition, it can be found from Figure 2f that an apparent drift of binding energy exists in 14 wt% SBO/SnO_2_, in comparison to pure SBO. This may be attributed to the change in internal electron density caused by the formation of the SBO/SnO_2_ heterojunction. Similar phenomena have been previously reported in the Bi_2_O_2_CO_3_/g-C_3_N_4_ heterostructured composite [18].

### 3.2. Morphology Analysis

**Figure 3:**
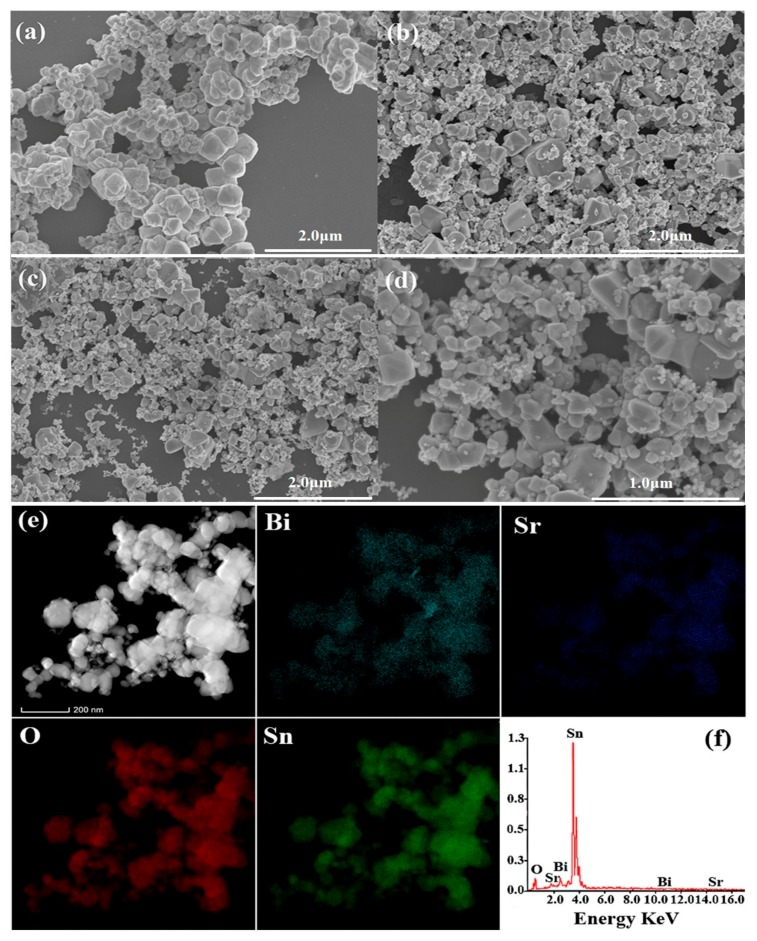
FESEM images of (**a**) SBO, (**b**) SnO_2_, (**c**) and (**d**) 14 wt% SBO/SnO_2_ composite, (**e**) Energy Dispersive Spectrometer (EDS) elemental mapping and (**f**) EDS spectrum of 14 wt% SBO/SnO_2_ composite.

The typical FE-SEM images of pure SBO, SnO_2_, and 14 wt% SBO/SnO_2_ composite, together with the EDS elemental mapping, are shown in Figure 3. As presented in Figure 3a, the pure SBO sample consists of large numbers of irregular blocks with sizes from about 0.1 to 0.4 μm. Figure 3b shows that the size distribution of SnO_2_ particles is wider and in the range of 0.04–0.6 μm, although the pure SnO_2_ sample has a similar morphology to SBO. It can be seen from Figure 3c,d that the size distribution of as-prepared SBO/SnO_2_ composite particles is also non-uniform. However, the sizes of SBO and SnO_2_ particles in SBO/SnO_2_ are markedly reduced after one-step hydrothermal synthesis and SnO_2_ particles with smaller size are strongly anchored on the surface of the SBO particles. The EDS elemental mapping of the 14 wt% SBO/SnO_2_ composite presented in Figure 3e further shows the distribution of SnO_2_ particles on the relatively larger SBO particles. The EDS spectrum of 14 wt% SBO/SnO_2_, shown in Figure 3f, confirms the peaks of Bi, Sr, O and Sn elements, indicating the existence of SBO and SnO_2_ components in the as-prepared composite.

**Figure 4:**
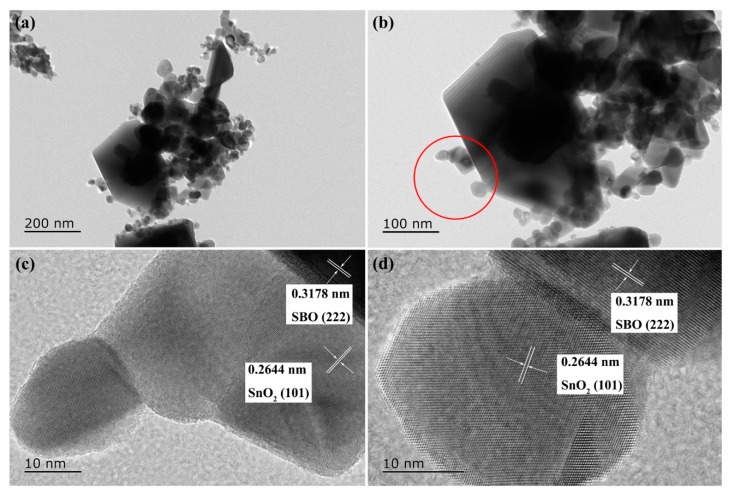
Transmission Electron Microscope (**a**,**b**) and High Resolution Transmission Electron Microscope (**c**,**d**) images of 14 wt% SBO/SnO_2_ composite.

TEM and high-resolution TEM (HRTEM) images were employed to further investigate the morphological structure of the 14 wt% SBO/SnO_2_ composite. As shown in Figure 4a,b, SnO_2_ particles are deposited randomly on the surface of SBO particles with an obviously larger size. HRTEM images presented in Figure 4c,d show that the clear lattice fringes with the interplanar distance of 0.3178 and 0.2644 nm corresponding to the (222) lattice plane of SBO and the (101) lattice plane of SnO_2_, respectively. Moreover, the distinguished interfaces between SBO and SnO_2_ are well built and contact tightly with each other, which strongly indicates the formation of the heterojunction between SBO and SnO_2_.

### 3.3. Optical Absorption Properties of SBO/SnO_2_ Catalysts

**Figure 5:**
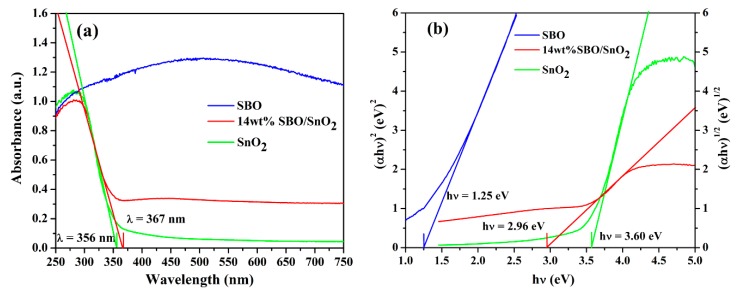
(**a**) UV-vis diffuse reflectance spectra and (**b**) the band gap energy of SBO, SnO_2_, and 14 wt% SBO/SnO_2_ composite.

Figure 5 exhibits the typical UV-vis diffuse reflectance spectra (DRS) of pure SBO, SnO_2_ and 14 wt% SBO/SnO_2_ composites. The absorption edge of SnO_2_ is at about 356 nm and can only absorb the UV light, while the UV-vis DRS of pure SBO shows a long absorption tail in the range of 400–800 nm due to a darker hue. Compared to pure SnO_2_, the UV-vis DRS of 14 wt% SBO/SnO_2_ has a redshift, and the corresponding absorption edge is about 367 nm. The band gap of as-prepared photocatalysts can be estimated by the formula, (*αhν*)*^n^* = A(*hν* − *E*_g_), where *h*, *α*, *E*_g_, *ν*, and A are the Plank constant, absorption coefficient, band gap, light frequency, and a constant, and the value of *n* depends on the type of optical transition in semiconductor. *n* = 2 is for direct transition and *n* = 1/2 is for indirect transition. The values of *n* for SBO and SnO_2_ are equal to 2, since they are direct semiconductors. The *E*_g_ of SBO and SnO_2_ estimated from the plot of (*αhν*)^2^, as a function of photon energy, are 1.25 and 3.60 eV, which are consistent with the previously reported values [24,30,34]. Considering the color of the prepared 14 wt% SBO/SnO_2_ composite, here we assume that it is an indirect semiconductor. The estimated *E*_g_ of 14 wt% SBO/SnO_2_ from the plot of (*αhν*)^1/2^ as a function of photon energy is about 2.96 eV, which indicates that, in the visible light region, the 14 wt% SBO/SnO_2_ composite has a stronger absorption ability than SnO_2_ and is favorable for photocatalytic reactions.

### 3.4. Photocatalytic Activity and Stability

**Figure 6:**
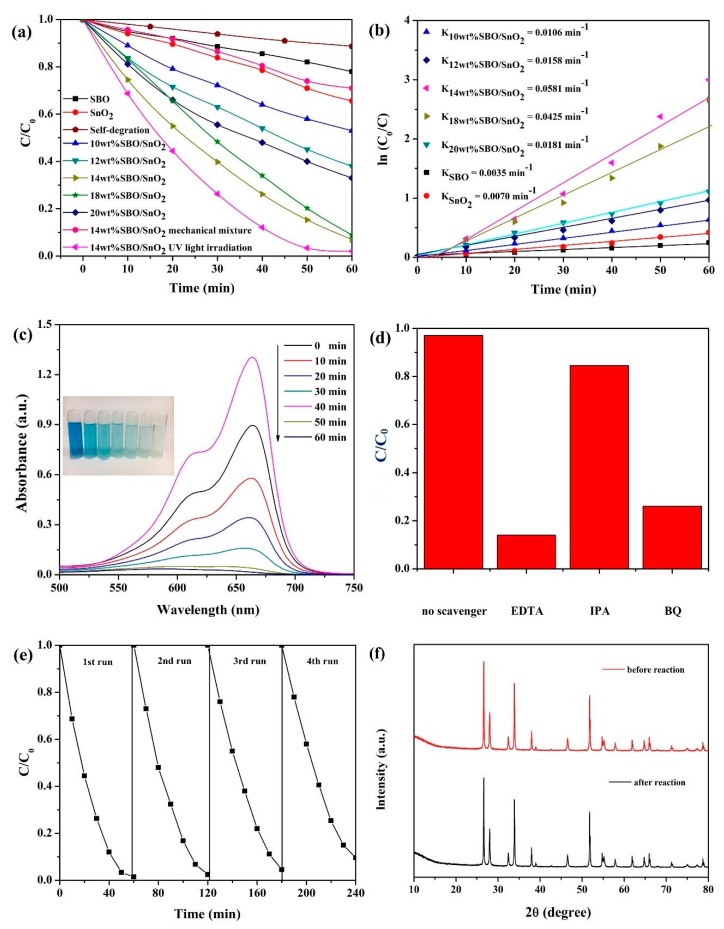
(**a**) Photodegradation of MB solution without photocatalyst and over the as-prepared SBO, SnO_2_, and SBO/SnO_2_ with different concentration of SBO; (**b**) kinetic plot of the photodegradation of MB solution versus irradiation time; (**c**) temporal absorption spectra of MB solution in the presence of 14 wt% SBO/SnO_2_; (**d**) degradation of MB over 14 wt% SBO/SnO_2_ in the presence of different scavengers; (**e**) cycling runs of 14 wt% SBO/SnO_2_ for MB degradation; and (**f**) XRD patterns of 14 wt% SBO/SnO_2_ before and after a four-cycle reaction.

The photocatalytic activities of SBO, SnO_2_ and SBO/SnO_2_ composites with different concentrations of SBO were investigated by the degradation of MB dye solution under the illumination of visible light (λ > 420 nm). As presented in Figure 6a, the blank test shows that, after 60 min reaction, no MB is removed without catalysts, indicating that the degradation of MB is driven by photocatalysis. Pure SBO and SnO_2_ show poor photocatalytic activity and can only degrade about 22.1% and 34.2% of MB after 60 min visible light irradiation, respectively. SBO/SnO_2_ composite catalysts obviously exhibit the increased photocatalytic efficiency of MB in comparison to pure SBO and SnO_2_. In particular, tge 14 wt% SBO/SnO_2_ composite displays the highest removal efficiency of MB (93.2%). However, the removal efficiency of MB is gradually decreased with the concentration of SBO beyond 14 wt%, because the excessive black SBO can probably screen the active sites on the surface of catalysts. Therefore, it is important to control the content of SBO in the composites in order to significantly enhance the photocatalytic performance. In addition, we can find that the degradation efficiency of MB over the 14 wt% SBO/SnO_2_ simple physical mixture is obviously slower than that of the composites. This strongly confirms the successful fabrication of SBO/SnO_2_ heterostructured photocatalysts in our current work. A comparative experiment on the degradation of MB over 14 wt% SBO/SnO_2_ under only the UV light illumination using a 300 W xenon lamp with the UVREF filter (λ < 400 nm) is also carried out. The measured results indicate that, under visible light illumination, the composite catalyst has only a slightly lower degradation efficiency than the same catalyst under UV light illumination. This clearly shows that the decomposition of MB, resulting from the visible light excitation, can be ignored. In addition, the degradation efficiency of MB over the 14 wt% SBO/SnO_2_ is comparable with other bismuth compounds or SnO_2_ based composites. For example, Yin et al. found that SnO_2_/BiVO_4_ composite catalysts showed remarkably improved visible-induced photocatalytic activity and the MB solution can be completely degraded under 120 min visible light irradiation [41].

Figure 6b shows that the photodegradation of the MB solution over pure SBO, SnO_2_, and SBO/SnO_2_ composites is the first-order reaction and the kinetics can be expressed using the following formula, −ln(*C*/*C*_0_) = *kt* (*C*_0_ is the concentration of dye after building the adsorption-desorption equilibrium, *C* is the reaction concentration at any time, *k* is the pseudo-first-order rate constant for degradation of dye, and *t* is the illumination time). It can be found that the value of *k* for MB degradation over 14 wt% SBO/SnO_2_ is about 0.0581 min^−1^ and is, respectively, 15.6 and 7.3 times higher than those of pure SBO and SnO_2_. Therefore, the results clearly show the obviously enhanced photocatalytic performance of SBO/SnO_2_ heterojunctions in comparison to pure SBO and SnO_2_. Figure 6c shows the temporal change of the absorption spectra in the degradation of MB dye over 14 wt% SBO/SnO_2_ within the time interval of 10 min under visible light irradiation. MB has a major absorption peak at around 664 nm. The peak intensity of MB, at around 664 nm, decreases to zero after 60 min irradiation, indicating the complete degradation of MB solution.

The free radical trapping experiments are further carried out to investigate the main types of reactive species like superoxide radical (•O_2_^−^), hydroxyl radical (•OH), and hole (h^+^) during the photodegradation of MB dye over SBO/SnO_2_ composites. In our work, the 5 mmol of benzoquinone (BQ), isopropanol (IPA), and ethylenediamine tetraacetic acid (EDTA) are used as the scavenger of •O_2_^−^, •OH, and h^+^, respectively. It can be found from Figure 6d that the degradation efficiency of the MB solution is obviously decreased when EDTA and BQ scavengers are added. This strongly indicates that the photoinduced •O_2_^−^ and h^+^ are the main reactive species in the photochemical reaction and play a crucial role for the degradation of MB. On the contrary, adding IPA almost does not suppress the degradation of MB solution, indicating that •OH is the secondary reactive species during the photochemical reaction.

The stability of photocatalysts is of great importance to their practical application. Figure 6e presents the cycling measurements, reflecting the long-term stability of 14 wt% SBO/SnO_2_ composite. It can be found that 14 wt% SBO/SnO_2_ does not show a distinct decrease in the photocatalytic activity after four cycles. As shown in Figure 6f, moreover, the crystal structure and phase composition of 14 wt% SBO/SnO_2_ do not change after a four-cycle photocatalytic reaction. Thus, we can see that the as-prepared SBO/SnO_2_ composites possess excellent structural stability under visible light illumination.

### 3.5. Possible Photocatalytic Mechanism

**Figure 7:**
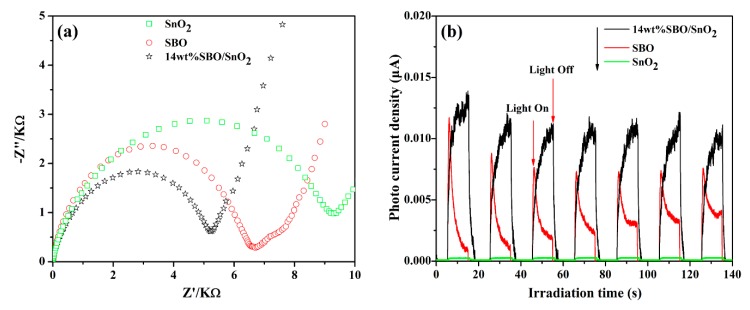
(**a**) Electrochemical impedance Spectroscopic plots and (**b**) transient photocurrent response of pure SnO_2_, SBO, and 14 wt% SBO/SnO_2_ heterojunction.

As clearly shown in Figure 6, the as-prepared SBO/SnO_2_ heterostructured composites exhibit higher visible light photocatalytic performance than pure SBO and SnO_2_. The EIS Nyquist plots and transient photocurrent responses of pure SBO, SnO_2_, and 14 wt% SBO/SnO_2_ composite are further measured to investigate the migration, transfer and recombination processes of photoinduced holes and electrons. It is a well-known fact that the Nyquist curve is a semicircle when the charge transfer is a decisive step. The relative size of the arc radius of Nyquist plots corresponds to the separation efficiency of light-excited carriers. Generally, the smaller the arc radius of the impedance spectrum is, the higher the separation efficiency of light-excited carriers is, and the faster the photocatalytic reaction is [42,43]. As presented in Figure 7a, the arc radius of EIS plot of 14 wt% SBO/SnO_2_ is obviously smaller than those of pure SBO and SnO_2_, which means that the as-synthesized SBO/SnO_2_ possesses a faster charge transfer process at the interface and more effective separation efficiency of photoinduced carriers than pure SBO and SnO_2_. The measured photocurrent responses of catalysts presented in Figure 7b further verify that SBO/SnO_2_ heterostructured composites are more effective in separating the photogenerated carriers, in comparison to pure SBO and SnO_2_, since the photocurrent density generated by SBO/SnO_2_ is obviously larger than that generated by pure SBO and SnO_2_.

**Figure 8:**
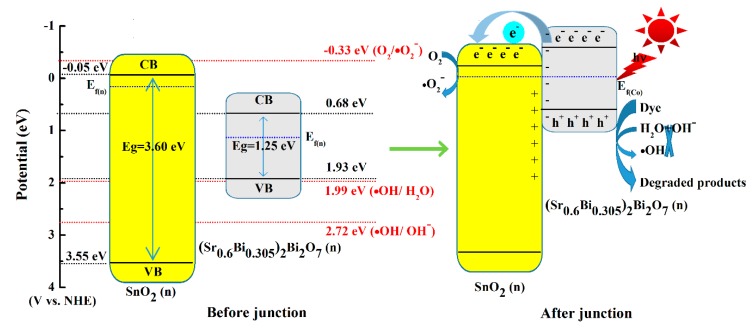
Possible mechanism of photocatalytic activity of SBO/SnO_2_ heterojunction for the degradation of MB under visible light irradiation.

Figure 8 schematically illustrates the possible mechanism of the photocatalytic degradation of MB by SBO/SnO_2_ composites. The enhanced photocatalytic activity of SBO/SnO_2_ composites could result from the charge transfer process between SBO and SnO_2_. The band edge position is the key to determining the separation of photoinduced charge carriers in the heterostructured composite. The band edge positions of SBO and SnO_2_ can be obtained from the following empirical formula [44]:

*E*_VB_ = 1/2*E*_g_ + *X* − *E*_e_ (1)

*E*_CB_ = *E*_VB_ − *E*_g_ (2)

where *E*_VB_ and *E*_CB_ are the valence and conduction band edge potentials, *X* and *E*_e_ represent the electronegativity of a semiconductor and the energy of free electrons (approximately 4.5 eV), respectively. The calculated values of *X* for SBO and SnO_2_ are 5.80 and 6.25 eV, respectively. Then the estimated *E*_CB_ and *E*_VB_ of SBO are 0.68 and 1.93 eV, and *E*_CB_ = −0.05 eV and *E*_VB_ = 3.55 eV are for SnO_2_. The CB of both SBO and SnO_2_ are more positive than the standard O_2_/•O_2_^−^ redox potential (−0.33 eV vs. NHE) [45], which indicates that the electrons excited in the CB of SBO or SnO_2_ cannot reduce O_2_ molecules into the strong oxidizing •O_2_^−^. The position of the Fermi level of n-type SBO is normally different from that of n-type SnO_2_. As shown in Figure 8, after the charge equilibrium is built, the Fermi level of SBO move up heavily and that of SnO_2_ move down slightly, and the n-n heterojunction is finally formed. Now the reduction of O_2_ into •O_2_^−^ becomes feasible, since the CB of SBO/SnO_2_ heterostructured composite will become more negative. Furthermore, the h^+^ photoinduced on the VB of SBO has strong oxidizing activity and can directly oxidize dye molecules. Considering the fact that the VB of SBO are more negative than the standard •OH/H_2_O redox potential (1.99 eV vs. NHE) [46], we can infer that the h^+^ on the VB of SBO cannot oxidize OH^-^ into •OH. Additionally, SnO_2_ cannot be excited to produce the holes and electrons under visible light illumination, owing to its relatively large *E*_g_ of about 3.60 eV. Based on the discussion above, we can ascribe the decoloration of the MB solution to the reaction with h^+^ and •O_2_^−^ during photocatalysis, which has been verified by the trapping experiments of the active species presented in Figure 6d. Therefore, the efficient photodegradation progress of MB over SBO/SnO_2_ composites can be described as follows,

SBO + *hν* (visible) → SBO (h^+^@VB + e^−^@CB) (3)

SBO (e^−^@CB) → SnO_2_ (e^−^@CB) (4)

SnO_2_ (e^−^@CB) + O_2_ → •O_2_^−^ (5)

h^+^ + •O_2_^−^ + dye → Degraded products (6)

## 4. Conclusions

(Sr_0.6_Bi_0.305_)_2_Bi_2_O_7_ (SBO) with the pyrochlore-type crystal structure belongs to one kind of special compound containing a small amount of vacancies and can be considered a potential semiconductor photocatalyst since it contains mixed valent states of Bi (Bi^3+^ and Bi^5+^) such as Bi_2_O_4_ with high photocatalytic degradation efficiency. In this work, SBO/SnO_2_ composites were first successfully synthesized via the facile one-step hydrothermal method. The characterizations of the phase structure, morphology, microstructure, and optical properties clearly showed the formation of heterojunction between SBO and SnO_2_ in the as-prepared composites. The fabricated SBO/SnO_2_ heterojunctions possessed the significantly enhanced photocatalytic performance towards MB degradation in comparison to pure SBO and SnO_2_. The trapping experiments showed that the photogenerated •O_2_^−^ and h^+^ are the major reactive species and play a vital role during the photodegradation of MB solution. The measured EIS and transient photocurrent responses demonstrated that the significantly enhanced catalytic efficiency of SBO/SnO_2_ nanocomposites is mainly attributed to the broad photoresponse range and efficient separation of photoinduced electron-hole pairs owing to the formation of heterojunction in the as-prepared composites.
